# Development of a Novel Magnetic-Bead-Based Automated Strategy for Efficient and Low-Cost Sample Preparation for Ochratoxin A Detection Using Mycotoxin–Albumin Interaction

**DOI:** 10.3390/toxins15040270

**Published:** 2023-04-05

**Authors:** Jin Ye, Hui Bao, Mengyao Zheng, Hongmei Liu, Jinnan Chen, Songxue Wang, Haihua Ma, Yuan Zhang

**Affiliations:** 1 Key Laboratory of Grain Information Processing and Control, Henan University of Technology, Ministry of Education, Zhengzhou 450001, China; 2 Henan Key Laboratory of Grain Photoelectric Detection and Control, Henan University of Technology, Zhengzhou 450001, China; 3 College of Information Science and Engineering, Henan University of Technology, Zhengzhou 450001, China; 4 Academy of National Food and Strategic Reserves Administration, Beijing 102600, China; 5 College of Food Science and Engineering, Henan University of Technology, Zhengzhou 450001, China

**Keywords:** ochratoxin A, albumin, mycotoxin–albumin interaction, automated magneto-controlled preparation, wine, beer

## Abstract

The mycotoxin ochratoxin A (OTA) is toxic to humans and frequently contaminates wine and beer. Antibodies are essential recognition probes for the detection of OTA. However, they have several drawbacks, such as high costs and difficulty in preparation. In this study, a novel magnetic-bead-based automated strategy for efficient and low-cost OTA sample preparation was developed. Human serum albumin, which is an economical and stable receptor based on the mycotoxin–albumin interaction, was adapted and validated to replace conventional antibodies to capture OTA in the sample. Ultra-performance liquid chromatography–fluorescence detection was used in combination with this preparation method for efficient detection. The effects of different conditions on this method were investigated. The recovery of OTA samples spiked at three different concentrations ranged from 91.2% to 102.1%, and the relative standard deviations (RSDs) were 1.2%–8.2% in wine and beer. For red wine and beer samples, the LODs were 0.37 and 0.15 µg/L, respectively. This reliable method overcomes the drawbacks of conventional methods and offers significant application prospects.

## 1. Introduction

Ochratoxin is a secondary metabolite of Aspergillus ochraceus, Aspergillus carbonarius, and Penicillium verrucosum [1]. Ochratoxin A (OTA) is the most toxic and common ochratoxin and can lead to severe nephrotoxic, carcinogenic, teratogenic, mutagenic, genotoxic, and immunotoxic effects [2,3,4]. The International Agency for Research on Cancer has classified OTA as a Group 2B potential human carcinogen [5]. OTA is predominantly found in cereals, grapes, beer, wine, dried fruit, cocoa, coffee, and other foods [6,7]. Beer and wine are widely consumed beverages and are among the largest sources of daily OTA intake in many countries [8,9]. Hence, many countries and regions have set very low maximum limits (MLs) of OTA to reduce the risk of OTA contamination [6]. For example, the MLs of OTA in wine, grape juice, and grape drinks are 2 µg/kg in the European Union (EU) [10], and the current national standard ML of OTA in wine and other alcoholic beverages in China (GB 2761-2017) is 2 µg/kg. Considering the significant adverse effects of OTA on human health, an accurate and efficient analytical method is essential to ensure that food products are safe from OTA contamination.

The currently used methods for OTA detection include high-performance liquid chromatography–fluorescence detection (HPLC-FLD) [11,12], ultra-high-performance liquid chromatography–fluorescence detection (UPLC-FLD) [13,14,15], or liquid chromatography–tandem mass spectrometry (LC-MS/MS) [16]. These are considered to be reliable and sensitive detection methods; however; actual samples cannot be analyzed without undergoing some preparation steps to eliminate interference from the complex matrix. Common preparation methods include solid-phase extraction (SPE) [17,18], immunoaffinity chromatography (IAC) [19,20,21], dispersive liquid–liquid extraction [22,23], QuEChERS [24,25], and matrix solid-phase dispersion (MSPD) [16,26]. OTA samples are most often prepared by IAC owing to its high selectivity and stability; however, the expensive and complex antibody preparation process and large differences between batches limit the application of IAC [27]. Thus, a novel sample preparation method is highly desirable. In particular, aptamers [28,29,30], molecularly imprinted polymers [31,32,33], and a novel receptor with antibody-like properties have been employed in applications ranging from separation systems to sensors.

Albumin is a water-soluble macromolecule with excellent biocompatibility. Many studies have been conducted on the interactions between xenobiotics and albumin. Poór M et al. calculated the binding constant of the OTA–albumin interaction using spectroscopic techniques and molecular modeling [34,35,36,37,38]. Human serum albumin has a higher binding constant (5.2 × 10^6^ M^−1^) [37], whereas that of bovine serum albumin (BSA) is slightly lower (3.2 × 10^5^ M^−1^) [38]. As these albumins are easy to obtain and their preparation cost is significantly lower than that of antibodies, albumin is a promising alternative to antibodies for the determination of OTA with a high affinity, low cost, and good biocompatibility. 

In this study, a novel magnetic-bead-based strategy for efficient and low-cost OTA sample preparation was developed using the mycotoxin–albumin interaction. An evaluation of the binding performance of OTA with HSA, BSA, and ovalbumin (OVA) revealed HSA as the optimal recognition probe, demonstrating its ability to replace antibodies for immobilization in magnetic beads. In addition, the entire sample preparation process was automatically completed via a magnetic-bead-based sample preparation platform developed in our previous study [15,39,40,41,42]. Coupled with ultra-performance liquid chromatography–fluorescence detection (UPLC-FLD), this novel method was successfully applied for the routine detection of OTA in beverages.

## 2. Results and Discussion

### 2.1. Adsorption Ability of the Three Types of Albumin–Magnetic Beads

**Figure 1:**
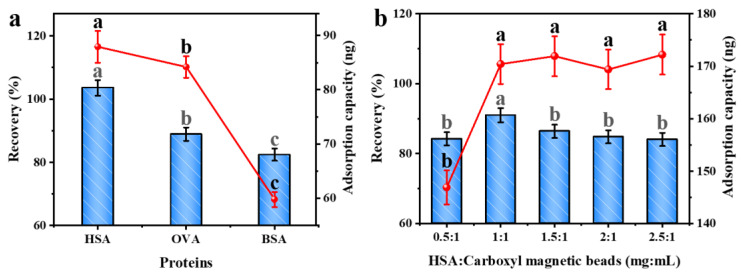
Screening for albumin (**a**); optimization of the ratio of magnetic beads to HSA (**b**). Blue bars represent recovery, and the red line represents the adsorption capacity. Letters denote different significant differences (ANOVA, *p* < 0.05).

The adsorption ability of different albumins, as identification probes, for OTA, was investigated by preparing three types of albumin–magnetic beads coupled with different albumins (HSA, BSA, and OVA). The recovery and maximum adsorption capacity of the albumin–magnetic beads for OTA were evaluated. The results show that the recovery of all albumin–magnetic beads is higher than 70% (Figure 1a) and that HSA has the strongest recognition ability for OTA, with a recovery >95%. In addition, the maximum adsorption capacity of HSA and OVA is >80 ng, whereas the maximum adsorption capacity of BSA is approximately 60 ng. Therefore, HSA is the optimal identification probe for albumins common to OTA. The adsorption ability of albumin in the magnetic beads was consistent with the binding constant of OTA–albumin in the solution.

### 2.2. Optimization of the Coupling Amount of HSA–Magnetic Beads

The coupling ratio of HSA and magnetic beads was optimized to achieve a high coupling efficiency of HSA with magnetic beads (Figure 1b). A series of ratios of HSA and magnetic beads (0.5:1, 1:1, 1.5:1, 2:1, and 2.5:1) was investigated. The maximum adsorption capacity of OTA was increased by increasing the coupling ratio from 0.5:1 to 1:1, indicating that the surface of the magnetic beads was not saturated. When the coupling ratio increased from 1:1 to 2.5:1, the adsorption capacity stabilized, indicating that the HSA on the magnetic bead surface was saturated. The recovery was the highest (>90%) when HSA and magnetic beads were coupled at a ratio of 1:1. Therefore, 1:1 was deemed the most suitable coupling ratio for HSA and magnetic beads.

### 2.3. The Principle of This Automated System

**Figure 2:**
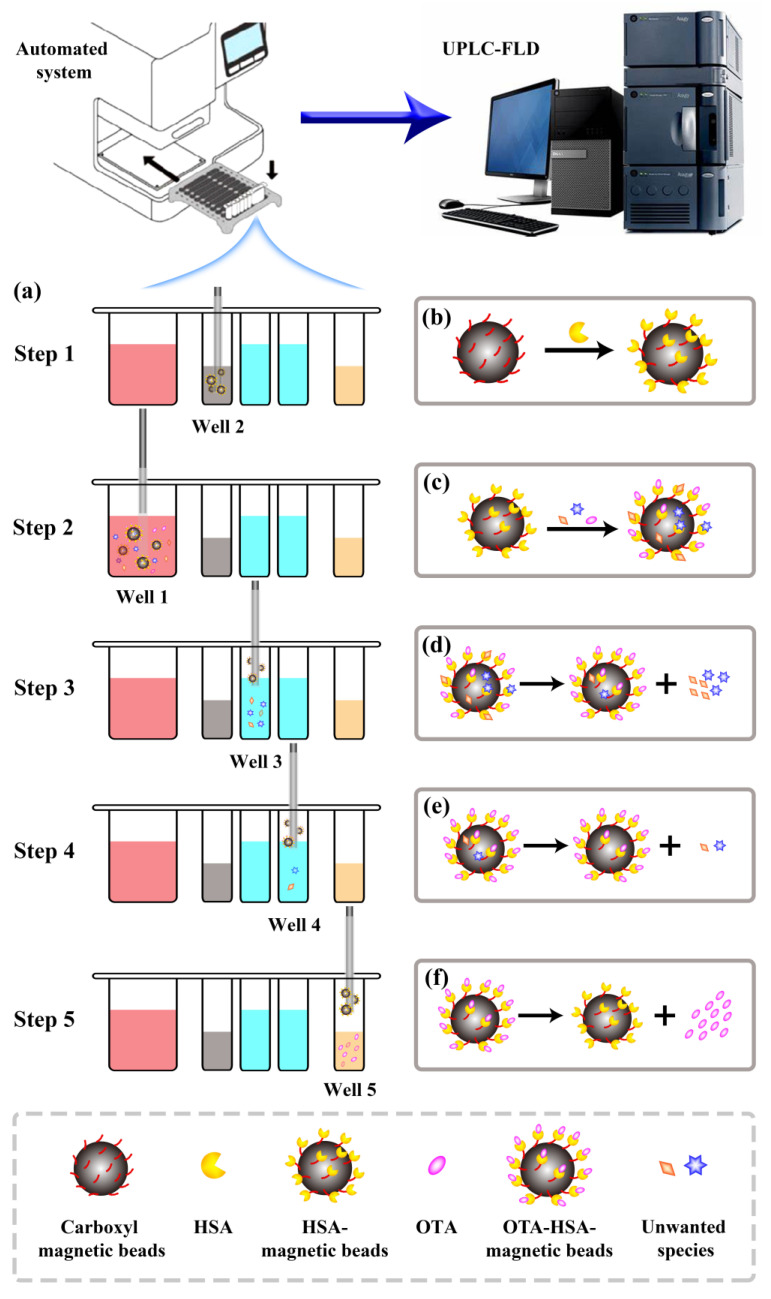
Clean-up procedure used by the automated system (**a**); synthesis of HSA–magnetic beads (**b**); HSA–magnetic beads capture OTA (**c**); removal of unwanted species (this step is performed twice) (**d**,**e**); release of OTA (**f**).

The principle of this automated system is illustrated in Figure 2. HSA was coupled to carboxylated magnetic beads via a condensation reaction between the carboxyl and amino groups to form HSA–magnetic beads (Step 1). OTA was then captured by the HAS–magnetic beads in the beer or wine sample to form OTA –HSA–magnetic beads (Step 2), which were transferred to the cleaning solution and washed twice to remove unwanted species (Steps 3 and 4). The OTA–HSA–magnetic beads were then transferred to the eluent using a magnetic stick and magnetic stick coat, after which the OTA was released into the eluent using organic reagents, forming an eluent solution containing only OTA (Step 5). Finally, UPLC-FLD was used to quantitatively detect the eluted OTA.

### 2.4. Optimization of the Sample Clean-Up Method

**Figure 3:**
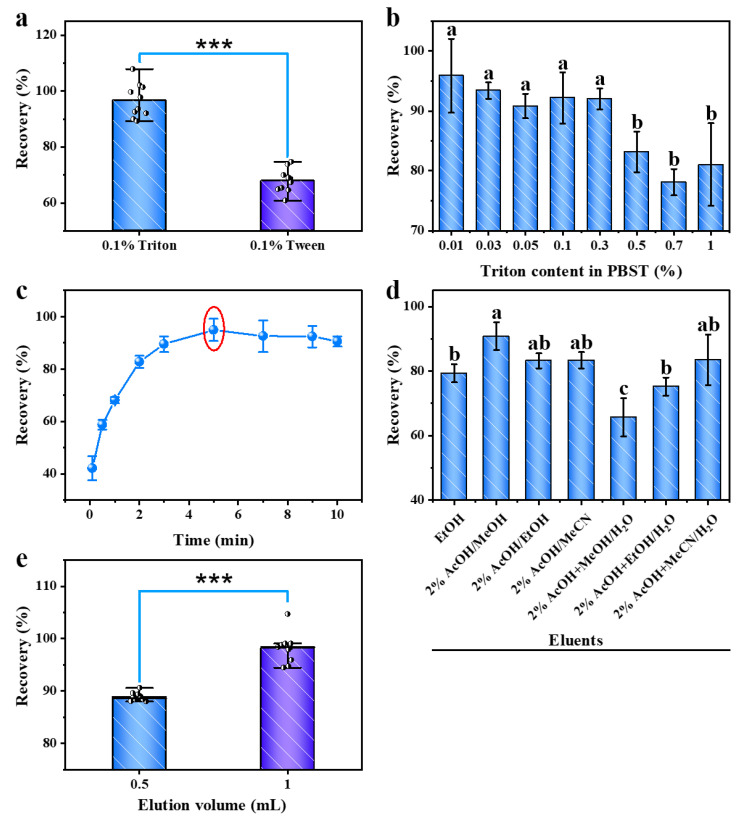
Optimization of the surfactant (**a**) and Triton content in PBST (**b**) in the reaction well (Well 1); optimization of the capture time (**c**) in the reaction well (Well 1); optimization of the eluate (**d**) and elution volume (**e**) in the elution/collection well (Well 5). Error bars were obtained from three parallel experiments and represent the standard deviation of recovery (as (**b**–**d**)). Black dots represent parallel data points (as (**a**,**e**)). *** Data are statistically different (ANOVA, *p* < 0.001). Letters represent different significant differences (ANOVA, *p* < 0.05). Red circles denote the optimal conditions.

To increase the contact area between the HSA–magnetic beads and sample and, thereby, improve the efficiency of OTA capture by HSA, the surfactant and its content were optimized (Figure 3a,b). Figure 3a shows that the recovery of PBST containing 0.1% Triton is above 95%, whereas the recovery of PBST containing 0.1% Tween is significantly different, at less than 80%. Therefore, Triton can promote the capture of OTA by HSA more effectively than Tween. Figure 3b shows that when the content of Triton in PBST is within 0.3%, the OTA recovery is higher than 90%. Therefore, the content of Triton in PBST can be controlled within 0.3%.

The reaction time was optimized to complete OTA detection in the shortest time. As shown in Figure 3c, the recovery of OTA gradually increases when the reaction time in the reaction well is increased. With a reaction time of 5 min, the recovery was closest to 1, whereas the recovery decreased slightly with longer reaction times. Therefore, 5 min was determined to be the optimal reaction time.

The eluate and elution volume were optimized to achieve the complete release of OTA, and the results are shown in Figure 3d,e, respectively. Based on Figure 3d, the OTA recovery was the highest when 2% acetic acid in methanol was used as the eluent. Therefore, 2% acetic acid in methanol was deemed the optimal eluent. Figure 3e shows that the recovery of OTA is close to 100% when the elution volume is 1 mL. Therefore, 1 mL was considered the optimal elution volume.

**Figure 4:**
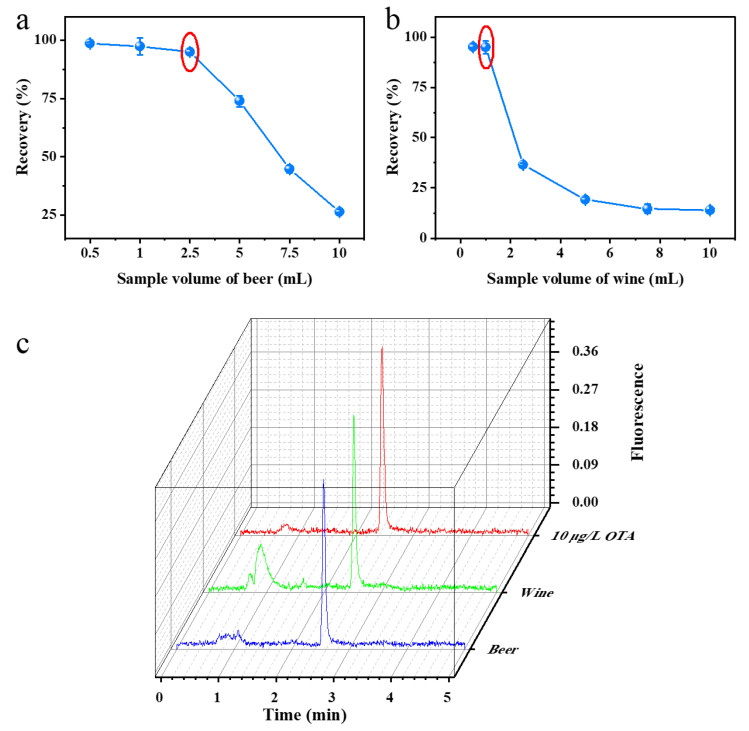
Optimization of the loading volume for beer (**a**) and wine (**b**); UPLC-FLD chromatograms of beer and red wine samples at optimal loading volume (**c**). Red circles indicate the optimal conditions.

Considering the issue of the alcohol tolerance of HSA (to avoid the mechanism interference effect), the loading quantities of beer and red wine samples were optimized prior to sample detection (Figure 4a,b). Figure 4a shows that the recovery gradually decreases with an increase in the beer loading volume. When the beer loading volume was 5 mL, the recovery was less than 75%. However, when the beer loading volume was 2.5 mL, the recovery was as high as 95%. Therefore, 2.5 mL was determined to be the maximum loading volume for beer. As shown in Figure 4b, the recovery gradually decreases with increasing red wine loading volume. When the red wine loading volume was 2.5 mL, the recovery was below 50%. However, when the beer loading volume was 1 mL, the recovery was as high as 95%. Therefore, 1 mL was determined to be the maximum loading volume for red wine. The retention time, peak shape, and peak area in the UPLC-FLD fluorescence spectra of the spiked samples of beer and red wine prepared under optimized conditions were essentially identical to those of the OTA standard solution of the same concentration (Figure 4c). Moreover, no peaks corresponding to impurities were observed in the vicinity of the target peak, which indicates the high purification efficacy of the technique.

### 2.5. Analytical Performance

The calibration curve was established by preparing a series of working solutions (0.125, 0.25, 0.5, 1.25, 2.5, 5, 10, and 20 ng/mL) to satisfy the requirements of an OTA analysis. The calibration curve (Figure S1) was constructed by plotting the peak areas (y) versus the injected concentrations of the standard solution (x), yielding the following regression equation: y = 4870.7x + 423.01 and a correlation coefficient (R^2^) of 0.9999, indicating excellent linearity.

The sensitivity of this method was estimated using the limit of detection (LOD) and limit of quantification (LOQ), which were calculated from the spiked blank samples at different concentrations of OTA. The two lowest concentrations, which produced 3-fold and 10-fold signal-to-noise (S/N) ratios, were considered to be the LOD and LOQ, respectively. The LOD and LOQ for red wine samples were 0.37 and 1.23 µg/L, respectively. For beer samples, the LOD and LOQ were 0.15 and 0.49 µg/L, respectively, which were lower than the MLs for OTA set for wine and other alcoholic beverages in China and the EU.

The specificity of HSA–magnetic beads for OTA was assessed by comparing the recovery of 13 common mycotoxins, including DON, 15-AcDON, 3-AcDON, 3G-DON, AFB_1_, AFB_2_, AFG_1_, AFG_2_, HT-2, NIV, ST, T-2, and ZEN, with that of OTA. The results are presented in Table 1. Among the common mycotoxins, all toxins except ST were not detected, and the recovery of ST was <1.5%, whereas the recovery of OTA reached 111.8%. These results indicated that HSA has good specificity for OTA.

The applicability of this method to real complex samples was investigated by preparing and analyzing real samples spiked at three different levels of OTA (low, medium, and high). The low-, medium-, and high-spiked recoveries of beer and red wine are listed in Table 2. The recoveries of beer and wine ranged from 91.2% to 102.1%, whereas the RSD was 1.2%–8.2%, indicating good accuracy and precision.

### 2.6. Detection of Actual Samples

Twenty-six brands of beer and wine, randomly purchased from supermarkets, were analyzed. As listed in Table 3, the amounts of OTA determined by this method were in good agreement with those obtained by the conventional IAC method (Figure S2, R^2^ = 0.9995), with no bias effect arising from the various types of sample matrices.

## 3. Conclusions

This study successfully established a novel magnetic-bead-based automated strategy for efficient and low-cost OTA sample preparation. The experimental data showed that the UPLC-FLD method has high sensitivity, accuracy, and precision. Although the binding capacity of HSA is weaker than that of conventional antibodies, further protein modification studies could improve its capacity for binding to OTA and other contaminants, leading to a low-cost receptor.

## 4. Materials and Methods

### 4.1. Experimental Materials

Human serum albumin (HSA), bovine serum albumin (BSA) and ovalbumin (OVA), Sodium azide, glycine, Tween-20, Triton X-100, and morpholinoethanesulfonic acid (MES) were purchased from Sigma-Aldrich (St. Louis, MO, USA). Carboxyl magnetic beads (10 mg/mL) were purchased from Kangyuan Techbio Biologicals (Beijing, China). Ultra-pure water used for the preparation of all the reagents was obtained from a Milli-Q purification system (Bedford, MA, USA). A standard solution of ochratoxin A (OTA), aflatoxin B_1_ (AFB_1_), aflatoxin B_2_ (AFB_2_), aflatoxin G_1_ (AFG_1_), aflatoxin G_2_ (AFG_2_), HT-2 toxin (HT-2), nivalenol (NIV), sterigmatocystin (ST), zearalenone (ZEN), T-2 toxin (T-2), Deoxynivalenol (DON), deoxynivalenol-3-glucoside (3G-DON), 3-acetyl-deoxynivalenol (3-AcDON), and 15-acetyl-deoxynivalenol (15-AcDON) were purchased from Biopure (Tulln, Austria) and were stored at −18 °C. Working solutions were prepared by dilution with HPLC-grade methanol and then stored in vials at 4 °C and renewed weekly. HPLC-grade methanol (MeOH) and Acetic acid (HAC) were purchased from Fisher Scientific (Atlanta, GA, USA).

### 4.2. Instrument and Analytical Conditions

Automated reaction conditions: an appropriate mixing frequency, mixing amplitude, and reaction time can ensure adequate reaction, washing, and elution of the samples. To prevent solvent spatter during mixing, the mixing amplitude was set to 80%. Other conditions, such as the reaction time, are listed in Table 4. At the end of the treatment, the eluate from the five wells was collected for UPLC-FLD testing.

UPLC-FLD analysis conditions: OTA terminal detection was performed using a Waters ACQUITY UPLC H-class system (Waters, Manchester, UK) consisting of a vacuum degasser, an auto-sampler, a binary pump, and a BEH reverse-phase C18 column (2.1 × 100 mm, 1.7 μm) for separation. The mobile phase consisted of acetic acid:water:methanol (1:51:48, *v*/*v*/*v*) at a flow rate of 0.3 mL/min. The excitation and emission wavelengths of the fluorescence detector were 333 and 460 nm, respectively. The column temperature was maintained at 40 °C; the sample temperature was maintained at 10 °C, and the sample injection volume was 10 μL. The Waters EMPOWER3 software 7.20.00.00 was used to acquire and analyze the data.

### 4.3. Coupling of Magnetic Beads to Albumin

First, 2 mg of protein was dissolved in 2 mL of MES to form a protein dilution. Magnetic beads (0.5 mL) were washed twice with absolute ethanol (0.5 mL) using a strong magnet. Next, 0.5 mL protein dilution was added, and the solution was incubated for 3 h. After magnetic separation, 1 mL of 2% glycine solution was added directly and blocked for 3 h. The solution was then washed twice with 1 mL 0.1% PBST and twice with 1 mL PBS. Finally, the volume was fixed in PBS (0.5 mL) and stored at 4 °C until further use.

### 4.4. Identification Ability of Magnetic Beads

The protein–magnetic beads were coupled with HSA, BSA, or OVA at ratios of 1 mg:1 mL. Subsequently, 50 µL of protein–magnetic beads were added to Well 2, and 4 ng (or 100 ng) of OTA standard was added to Well 1 and diluted to 5 mL with PBST. After processing using an automated magneto-controlled preprocessing platform, the eluent in Well 5 was collected for detection via HPLC-FLD.

### 4.5. Optimization of Conditions

#### 4.5.1. Optimization of the Protein/Magnetic Bead Coupling Ratio and Loading Volume

The magnetic beads (0.1, 0.2, 0.3, 0.4, and 0.5 mL) were coupled with 0.2 mg of HSA to form HSA–magnetic beads and added to Well 2. Subsequently, 4 ng (or 200 ng) of OTA standard was added to Well 1 and diluted to 5 mL with PBST. After processing using an automated magneto-controlled preprocessing platform, the eluent in Well 5 was collected for detection via HPLC-FLD. The optimum ratio of protein-to-magnetic beads was investigated. HSA–magnetic beads (50, 100, and 200 µL; HSA:magnetic beads = 1 mg:1 mL) were then collected, and 4 ng of OTA was added to Well 1 and eluted with 1 mL of 2% acetic acid in methanol. The effect of the loading volume of the HSA–magnetic beads on recovery was then investigated.

#### 4.5.2. Optimization of the Elution Solvent and Volume

HSA–magnetic beads (100 µL) were added to Well 2, and 8 ng of OTA was added to Well 1 and eluted with different solvents to investigate the optimal eluent of HSA–magnetic beads. HSA–magnetic beads (50 µL) were added to Well 2, and 4 ng of OTA was added to Well 1, and the eluent volume of HSA beads was investigated with different volumes of 2% acetic acid in methanol.

#### 4.5.3. Optimization of the Sample Extract

OTA (8 ng) was added to Well 1 and then diluted to 5 mL with a PBST solution containing 0.1% Tween and 0.1% Triton. Finally, 1 mL of 2% acetic acid in methanol solution was eluted to determine the optimal surfactant. The OTA in Well 1 was diluted to 10 mL with PBST solutions containing different surfactant concentrations, and the other conditions were maintained unchanged to investigate the optimal proportion of surfactant. Similarly, 8 ng of OTA was added to Well 1 and diluted with a PBST solution containing 0.1% Triton to 5, 7, 8, 9, and 10 mL. The effect of different volumes of the reaction solution on the OTA recovery was investigated.

#### 4.5.4. Optimization of the Capture Time

The mixing time in Well 1 was set to 0.1, 0.5, 1, 2, 3, 4, 5, 7, and 9 min, and the other conditions were maintained unchanged. The effect of different capture times on the OTA recovery was investigated.

#### 4.5.5. Optimization of the Beer Loading Volume

OTA (10 ng) was added to Well 1, and 2.5, 3, 3.5, 4, 4.5, and 5 mL of beer were added. When the volume was <10 mL, the beads were diluted to 10 mL with PBST, while the other conditions remained unchanged. The ability of the HSA–magnetic beads to tolerate beer matrices was also investigated.

#### 4.5.6. Optimization of the Loading Volume of Red Wine Samples

OTA (10 ng) was added to Well 1, and 0.5, 1, 2.5, 5, 7.5, and 10 mL red wine were then added. When the volume was <10 mL, the beads were diluted to 10 mL with PBST, while the other conditions remained unchanged. The ability of the HSA–magnetic beads to tolerate red wine matrices was also investigated.

### 4.6. Sample Treatment

The recovery of beer and red wine samples was determined using low, medium, and high spikes (final OTA concentrations of 1, 2, and 4 µg/L in the samples), and the other conditions were unchanged.

### 4.7. Investigation of the Detection Performance

The linearity was determined using eight concentrations of OTA (0.125, 0.25, 0.5, 1.25, 2.5, 5, 10, and 20 µg/L), and the regression equation and coefficient (R^2^) were calculated by plotting the peak area (y) against the concentration of OTA (x). The LOD and LOQ were estimated in triplicate using a serially diluted OTA standard solution, which produced a chromatogram peak with S/N ratios of 3 and 10, respectively. The selectivity was investigated using 13 common mycotoxins: DON, 15-AcDON, 3-AcDON, 3G-DON, AFB_1_, AFB_2_, AFG_1_, AFG_2_, HT-2, NIV, ST, T-2, and ZEN.

### 4.8. Manual IAC Clean-Up Procedure

The following IAC clean-up procedures were based on the appropriate ISO standard [43] with some modifications. In particular, an aliquot (4 mL) of the sample solution extracted from the obtained sample was diluted with the PBST solution (26 mL). The diluted extract was centrifuged at 8000× *g* for 5 min before the conduction of the IAC of OTA at a flow rate of approximately 1–2 drops/s and thereafter washed with a solution containing PBST (10 mL) and distilled water (10 mL) at a flow rate of 2 drops/s. After elution, the OTA eluted with the elution solvent (1 mL) was transferred to an LC vial.

## Figures and Tables

**Table 1:** Comparison of the recovery of 13 common mycotoxins with that of OTA.

Toxins	Spiking Concentration (µg/L)	Recovery (%)	RSD (%)
DON	30.00	ND	ND
15-AcDON	4.00	ND	ND
3-AcDON	8.00	ND	ND
3G-DON	5.00	ND	ND
AFB_1_	0.20	ND	ND
AFB_2_	0.20	ND	ND
AFG_1_	0.20	ND	ND
AFG_2_	0.20	ND	ND
HT-2	2.00	ND	ND
NIV	40.00	ND	ND
ST	0.20	1.1	0.2
T-2	0.16	ND	ND
ZEN	4.00	ND	ND
OTA	1.00	111.8	0.04

**Table 2:** Recovery of beer and wine at different spiking levels (*n* = 3) (RSDs: relative standard deviations).

Spiked Level (µg/L)	Beer		Wine	
	Average Recovery (%)	RSD (%)	Average Recovery (%)	RSD (%)
1	101.4	8.2	102.1	2.9
2	91.2	2.9	96.4	2.2
4	97.0	1.2	100.4	2.7

**Table 3:** Results of real wine and beer sample test.

Brand	Concentrations (µg/L)	
	This Method	Reference IAC Method
RIO 3° Cocktail White Peach Brandy Beer	ND	ND
Qingyi Pineapple Beer	ND	ND
Corona Beer	ND	ND
Brave Rudolf Rock Beer	0.16	0.16
Kaiser Yellow Beer	ND	ND
Tsingtao Augerta Beer	ND	ND
Kingway King Of Golden Wheat Beer	ND	ND
Qingdao Bingchun Beer	ND	ND
Tsingtao Laoshan Beer	ND	ND
Barreker Wheat Beer	ND	ND
Qingyi Wheat King Beer	ND	ND
Tsingtao Whole Wheat Whitebeer	ND	ND
Hoegaarden Whitebeer	ND	ND
Hoegaarden Amber Orange Beer	ND	ND
Budweiser Blackgold Beer	ND	ND
Budweiser Supreme Beer	ND	ND
Yanjing U8 Beer	ND	ND
Beijing Yanjing Brewery	ND	ND
Yanjing Boutique Beer	0.26	0.20
Weijixiong Craft Beer	ND	ND
Harbin Beer Chunshuang Pale Lager	0.17	0.26
Hoegaarden Rosee Beer	ND	ND
Fort Shengfei Reserve Dry Red Wine	1.25	1.35
Changbai Mountain Ice-Red Wine	ND	ND
Home-made Red wine (1)	7.89	7.56
Home-made Red wine (2)	3.26	3.17

**Table 4:** The sequence of events in an automated clean-up program.

Step	Well	Mixing Time/min	Mixing Frequency/Hz	Volume/mL
Transfer	2	1.0	6.5	0.8
Reaction	1	5.0	1.5	10.0
Wash 1	3	1.0	6.5	1.0
Wash 2	4	1.0	6.5	1.0
Elution/Collection	5	1.0	6.5	1.0

## Data Availability

Not applicable.

## Supplementary Materials

The following supporting information can be downloaded at: https://www.mdpi.com/article/10.3390/toxins15040270/s1, Figure S1. The calibration curve, Figure S2. Comparison on the determination of OTA in real samples between this method and IAC.
