# Supplementary material for: Development of a Novel Magnetic-Bead-Based Automated Strategy for Efficient and Low-Cost Sample Preparation for Ochratoxin A Detection Using Mycotoxin–Albumin Interaction
Source: Toxins (Basel). 2023 Apr 5;15(4):270. doi: 10.3390/toxins15040270 (PMC10145472; doi:10.3390/toxins15040270)
Supplement: Supplementary file 1 [file toxins-15-00270-s001.zip › toxins-2257756-supplementary.pdf]

Supplementary Materials

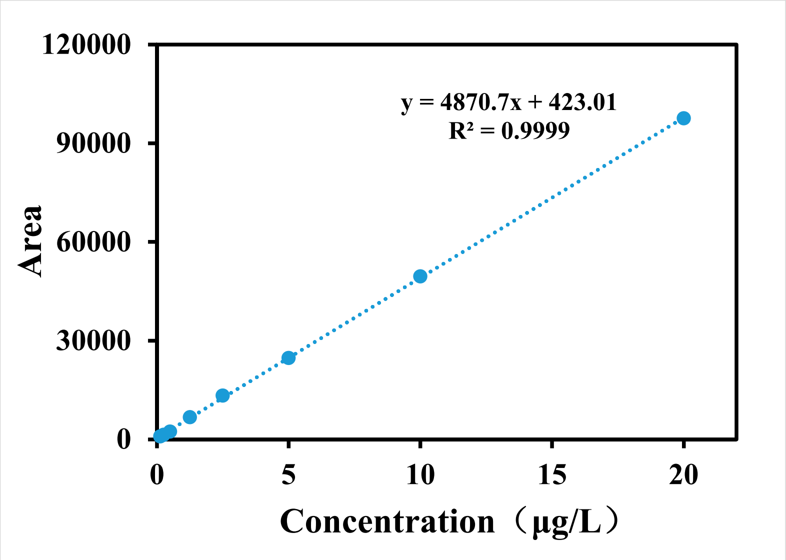

Figure S1. The calibration curve

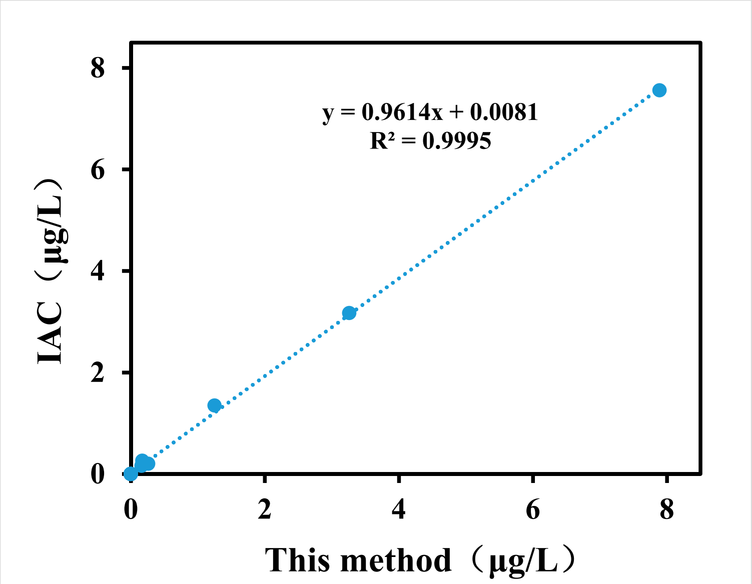

Figure S2. Comparison on the determination of OTA in real samples between this method and IAC.
